# Effect of Mobile Application-Based Cognitive Behavioural Therapy on Atopic Dermatitis Comorbid With Obsessive-Compulsive Disorder: A Retrospective Study

**DOI:** 10.62641/aep.v54i4.2300

**Published:** 2026-08-15

**Authors:** Dan Liang, Lihong Yang, Xian He

**Affiliations:** ^1^Department of Allergy, Chengdu First People’s Hospital, 610041 Chengdu, Sichuan, China

**Keywords:** cognitive behavioural therapy, mobile application, telemedicine, atopic dermatitis, obsessive-compulsive disorder, comorbidity

## Abstract

**Background:**

This study aimed to evaluate the effect of mobile application-based cognitive behavioural therapy (MCBT) in patients with atopic dermatitis (AD) and comorbid obsessive-compulsive disorder (OCD).

**Methods:**

This retrospective study analysed 100 patients with AD and comorbid OCD treated at Chengdu First People’s Hospital from January 2024 to December 2025. Patients were divided into an MCBT group (n = 50) and a conventional group (n = 50) according to the treatment they received. All patients received routine medication. The conventional group received conventional cognitive behavioural therapy (CBT), whereas the MCBT group received MCBT via mobile applications. Treatment duration was 12 weeks for both groups. The Eczema Area and Severity Index (EASI), Yale–Brown Obsessive Compulsive Scale (Y-BOCS), Dermatology Life Quality Index (DLQI), and treatment adherence were compared between the two groups before and after treatment.

**Results:**

After 12 weeks of treatment, EASI, Y-BOCS, and DLQI scores significantly reduced from baseline in both groups (all *p* < 0.01). Moreover, the MCBT group had significantly lower EASI [10.5 (3.7, 18.6)], Y-BOCS (10.2 ± 2.1), and DLQI (5.3 ± 1.8) scores than the conventional group [14.4 (6.8, 26.9), 15.5 ± 2.3, 10.6 ± 2.7] (all *p* < 0.01). The overall treatment adherence rate was significantly higher in the MCBT group (82.0%) than in the conventional therapy group (64.0%; χ^2^ = 4.110, *p* = 0.042).

**Conclusions:**

MCBT combined with conventional therapy may alleviate dermatological and obsessive-compulsive symptoms in patients with AD and OCD, while also improving treatment adherence and quality of life. Given its simplicity and feasibility, further large-scale prospective studies are warranted to validate its clinical value.

## Introduction

Atopic dermatitis (AD), also known as atopic eczema, is a chronic, recurrent, inflammatory skin disease characterized by dry skin, eczematous lesions and severe itching. Its aetiology is closely linked to genetics, immune dysfunction, impaired skin barrier function, environment, and psychological factors [1]. The chronic and recurrent nature of AD seriously affect patients’ physical and mental health. It is known that the skin and nervous system differentiate from the same ectoderm and are interconnected through the skin–brain axis. Persistent pruritus, repetitive scratching, and chronic inflammation in AD may influence emotional regulation and cognitive processing, thereby increasing the risk of psychiatric comorbidities. Many patients with chronic skin diseases have comorbid mental illnesses, among which obsessive-compulsive disorder (OCD) is relatively common [2]. A large-sample case-control study showed that patients with AD have a twofold higher risk of comorbid OCD than individuals without AD (OR = 2.00, 95% CI 1.5–2.7), imposing a double disease burden [3].

Patients with AD and comorbid OCD may exhibit repetitive scratching and excessive cleaning behaviours, which aggravates itching and skin lesions, forming a vicious cycle [4]. Such patients tend to seek care in dermatology clinics, where their obsessive-compulsive symptoms are often underdiagnosed. Although topical corticosteroids can alleviate skin symptoms, it is difficult to break the vicious cycle, leading to a prolonged disease course and high relapse rate [5]. Antipsychotic medications for OCD usually have a slow onset of action and many adverse reactions, and clinical practice often requires combining them with psychotherapy [6].

Cognitive behavioural therapy (CBT) is a psychotherapy based on cognitive and behavioural theories. It aims to identify and correct patients’ irrational cognitions, change maladaptive behaviour patterns and alleviate negative emotions. CBT is a first-line treatment for OCD; however, it typically requires in-person sessions at healthcare facilities, which may be less effective for patients receiving home-based care for dermatitis [7]. With the advancement of “Internet plus” medical service, remote management using mobile devices and applications is increasingly used for outpatient treatment of chronic diseases [8]. Nevertheless, reports on mobile application-based cognitive behavioural therapy (MCBT) for patients with AD and OCD are scarce. Therefore, this study included 100 patients with AD and comorbid OCD admitted to our hospital from January 2024 to December 2025 and compared the treatment effects of conventional CBT and MCBT, aiming to provide a reference for clinical treatment.

## Materials and Methods

### General Information

This retrospective study included 100 patients with AD and OCD treated at the Department of Allergy, Chengdu First People’s Hospital, between January 2024 and December 2025. Based on the actual treatment they received, patients were divided into an MCBT group and a conventional group, with 50 patients in each. Grouping method: During the study period (2024–2025), MCBT was sequentially administered to patients capable of smartphone use who provided informed consent for remote management. Patients who did not receive MCBT continued to receive conventional CBT.

The inclusion criteria were as follows: (1) meeting the diagnostic criteria for AD according to the Chinese Guidelines for the Diagnosis and Treatment of Atopic Dermatitis (2020 edition) [9], including eczematous lesions and pruritus, disease duration >6 months, and elevated serum IgE levels (>200 IU/mL) or peripheral blood eosinophil (>5%); (2) meeting Diagnostic and Statistical Manual of Mental Disorders, Fifth Edition (DSM-5) for OCD [10] with primary clinical manifestations of skin-related obsessive thoughts and behaviours (e.g., such as scratching, skin picking and excessive cleaning) and a Yale–Brown Obsessive Compulsive Scale (Y-BOCS) [11] score ≥16; (3) age 18–60 years; (4) disease duration ≥6 months; (5) normal cognitive function and ability to own and proficiently use a smartphone; and (6) complete clinical data available.

The exclusion criteria were as follows: (1) comorbid other skin diseases (e.g., psoriasis, contact dermatitis); (2) comorbid severe physical illnesses or other mental diseases (e.g., schizophrenia, bipolar disorder); (3) receiving pharmacological treatment for OCD (e.g., antidepressants, antipsychotics, or other psychotropic medications) during the study period; (4) pregnancy or breastfeeding; or (5) inability to complete treatment and follow-up.

This study was approved by the Ethics Committee of Chengdu First People’s Hospital (approval number: 2026-IIT-029). The study protocol complied with the ethical requirements of the Declaration of Helsinki. All data were anonymized before statistical processing. Written informed consent was obtained from all patients.

### Treatment methods

#### Conventional Group

Patient received standard pharmacotherapy and conventional CBT for 12 weeks.

(1) Pharmacotherapy: For mild skin lesions (Eczema Area and Severity Index [EASI] ≤7 points [12]), topical 0.1% tacrolimus ointment (Sichuan Mingxin Pharmaceutical Co., Ltd., Chengdu, Sichuan, China; batch NO. H20123430) was applied once or twice a day; for moderate to severe skin lesions (EASI >7 points), topical mometasone furoate cream (Heng’an Fulin Pharmaceutical Co., Ltd., Yichang, Hubei, China; batch NO. H20074173) was applied once or twice a day, with gradual dose tapering and discontinuation after symptom relief; for severe itching, oral loratadine tablets (Sanmenxia Sanovi Pharmaceutical Co., Ltd., Sanmenxia, Henan, China; batch NO. H20020174) 10 mg once a day were given. No antiobsessive or antipsychotic medications were prescribed during the study period. All OCD symptoms were managed exclusively with CBT-based psychotherapy.

(2) CBT: Conventional CBT was delivered in the outpatient setting once weekly for 50 minutes.

(a) Cognitive guidance: Patients were educated about AD and OCD, including that AD is an allergic condition associated with immune system overactivation, and that compulsive behaviours such as scratching, skin picking, and excessive cleaning can damage the epidermis and worsen the disease. Patients were also informed that corticosteroids and antipsychotic medications must be used as prescribed; unauthorized discontinuation or dose adjustment may not only reduce efficacy but also lead to drug resistance and adverse effects.

(b) Behavioural guidance: Patients were instructed to establish regular skin care routine, maintain skin cleanliness and hydration, follow a balanced diet and avoid allergens exposure. They were encouraged to use distraction techniques (e.g., listening to music, watching videos), meditation, and competing responses (e.g., fist clenching, deep breathing, squeezing a stress ball, gently tapping the skin) to reduce obsessive thoughts and behaviours, thereby breaking the “itch–scratch–lesion exacerbation” cycle.

#### MCBT Group

The MCBT group received MCBT treatment in addition to standard pharmacotherapy for 12 weeks, once weekly, 50 minutes per session. Treatment followed a standardized framework initial assessment (week 1–2), cognitive and behavioural transformation (week 3–10), and consolidation (week 11–12). Individualized treatment strategies were developed according to patients’ dominant symptoms and behavioural patterns. The treatment details are as follows:

(1) Platform development and MCBT team formation: A telemedicine platform was established using the hospital’s official WeChat account, WeChat mini-programs, and WeChat groups. The official account provided basic medical services (e.g., appointment scheduling, health education content dissemination, report access and online payment). Health education materials were delivered twice weekly through the official account using illustrated formats with supplementary video explanations. The WeChat mini-program included disease management modules such as online consultations, patient follow-up, medication calendar tracking and AI-based automated responses. Patients were required to upload medication records and symptom changes weekly. The WeChat group served as a real-time interactive channel for communication between healthcare providers and patients, as well as among patients.

The MCBT team consisted of two allergists, two psychiatrists and four nurses from each department. The team collaboratively developed health education materials, which were uploaded to the above mobile platforms in illustrated formats with video explanations. Team members took daily online duty in the WeChat group, with two members available daily, and the duty schedule was announced within the group.

(2) Initial assessment and platform onboarding (week 1–2): At the first outpatient visit, a multidisciplinary consultation was conducted, during which dermatologists, allergists and psychiatrists jointly assessed disease severity. Patients were instructed to follow the hospital’s official WeChat account, download the WeChat mini-program, and join the AD–OCD WeChat group. Assistance was provided to establish personal electronic health records, and detailed instructions were given on how to use each mobile application. For privacy protection, all patients registered using hospital medical record numbers, and personal information uploaded via the WeChat platform was accessible only to authorized medical staff. Additionally, patients were advised not to share private information in group communications.

Patients completed the Y-BOCS at the first visit. In addition, an internally developed questionnaire assessing disease perception and self-management in patients with AD was administered as an auxiliary tool to identify patient-specific problems (e.g., low self-esteem related to skin lesions, excessive attention to pruritus, compulsive scratching and excessive cleaning), and individualized MCBT strategies were developed. The questionnaire was not an outcome measure and was therefore excluded from statistical analysis.

(3) Remote health guidance and dynamic CBT: (a) During week 3–10, patients received standardized remote CBT therapy. Team members interacted with patients daily via WeChat groups, reminding them to adhere to medical advice and medication, diligently study the disease-related educational materials pushed by the official WeChat account, and record skin symptoms, compulsive behaviours, and medication usage on the mini-program. Medical staff can view uploaded data in real time; if abnormalities were detected, they contacted the patient in a timely manner to understand the cause. For patients whose condition worsened, prescriptions were adjusted promptly; for those with poor adherence, communication frequency was increased, and family members were involved in supervising the patient’s behaviour. (b) Weekly video consultations were conducted, with multidisciplinary physicians performing remote assessments of skin lesion recovery and gaining deeper insight into the patient’s psychological state, cognitive patterns, and behavioural habits to reinforce treatment adherence. After discharge, patients completed a weekly questionnaire and upload it via WeChat, allowing medical staff to adjust the treatment plan in a timely manner based on disease trends. (c) As a real-time communication tool, WeChat groups allowed patients to ask questions at any time. MCBT team members on duty answered questions promptly and affirmed patient progress to enhance confidence. At the same time, patients were encouraged to exchange recovery experiences in the WeChat group, share techniques to overcome skin lesions and correct compulsive behaviours, and build confidence through mutual encouragement and enhanced communication.

(4) Consolidation and relapse prevention (week 11–12): Emphasis was placed on relapse prevention and self-management. For patients whose condition improved, dosage was gradually reduced, but medication was not stopped immediately to avoid rebound of the therapeutic effect. Recovered patients were encouraged to retain their membership in the WeChat group, continue receiving antiallergy treatment and healthy lifestyle guidance, and share their recovery experience as “successful individuals”. The MCBT group regularly summarized cognitive and behaviour methods that patients had found effective in daily life, compiled them into standardized guidance content, and uploaded them to the WeChat official account, forming a reusable rehabilitation resource library for subsequent patients.

### Observation Indicators

#### Baseline Characteristics

Baseline characteristics, data on sex, age, disease duration, AD severity, marital status, educational level, and employment status were collected from the hospital’s electronic medical records.

#### AD Symptoms

AD symptoms were assessed using the EASI [12] at baseline and after 12 weeks of treatment. The body was divided into four regions: head/neck, trunk, upper limbs, and lower limbs. Four clinical signs—erythema, induration/papulation, excoriation, and lichenification—were assessed, each scored from 0 to 3. The score for each region = symptom score × area weight × proportion of skin involvement. The total EASI score is the sum of the four regions scores, ranging from 0 to 72; higher scores indicate greater disease severity. EASI severity categories were defined as follows: 0 = clear/no eczema; 0.1–1.0 = almost clear; 1.1–7.0 = mild; 7.1–21.0 = moderate; 21.1–50.0 = severe; 50.1–72.0 = very severe.

#### Obsessive-Compulsive Symptoms

Obsessive-compulsive symptoms were assessed using the Y-BOCS at baseline and after 12 weeks [11]. The scale includes two dimensions—obsessions and compulsions—with 10 items, each scored from 0 to 4, yielding a total score ranging from 0 to 40. Higher scores indicate more severe symptoms. A Y-BOCS score ≥16 indicated clinically significant OCD [13].

#### Quality of Life

Quality of life was assessed using the Dermatology Life Quality Index (DLQI) at baseline and after 12 weeks [14]. The DLQI has 10 items covering symptoms and feelings, psychological impact, daily activities, clothing, social and leisure activities, physical exercise, work or study, interpersonal relationships, sexual activity and treatment burden. Each item scored from 0 to 3, with a total score ranging from 0 to 30. Higher scores indicate poorer quality of life.

#### Treatment Adherence

Treatment adherence was assessed after 12 weeks using the 8-item Morisky Medication Adherence Scale (MMAS-8) [15]. The questionnaire covers forgetting medication, non-adherence not due to forgetfulness, intentional non-adherence, missing medication during travel, overall recent adherence, discontinuation after symptom improvement, discontinuation due to cost, and frequency of forgetting.

Adherence was categorized as follows: high (8 points), moderate (6–7 points), and low (<6 points). Overall adherence rate = (number of patients with high adherence + number with moderate adherence) / total number of patients × 100%.

### Statistical Analysis

Statistical analyses were assessed using SPSS version 22.0 (IBM Corp., Armonk, NY, USA). Normality of continuous variables was assessed with the Shapiro–Wilk test. Normally distributed data are expressed as mean ± standard deviation (SD); within-group comparisons used the paired t-test, and between-group comparisons used the independent samples t-test. Non-normally distributed data are expressed as median and interquartile range [M (Q1, Q3)] and were compared using the Mann–Whitney U test; within-group differences were compared using the Wilcoxon signed-rank test. Categorical variables are expressed as percentages (%) and were compared using the chi-square (χ^2^) test. A two-sided *p* value < 0.05 was considered statistically significant.

## Results

### Baseline Characteristics

No significant differences were observed between the two groups in sex, age, disease duration, AD severity, marital status, educational level, or employment status (*p* > 0.05), indicating comparability between the groups (Table 1).

**Table 1.** **Comparison of baseline data between the two groups**.

Clinical characteristics		Conventional group (n = 50)	MCBT group (n = 50)	χ^2^/t	*p*
Sex				0.040	0.841
	Male	24 (48.0)	25 (50.0)		
	Female	26 (52.0)	25 (50.0)		
Age (years)				0.472	0.638
	Range	18–58	19–59		
	Mean ± SD	38.6 ± 6.2	39.2 ± 6.5		
Marital status				0.457	0.499
	Married	35 (70.0)	38 (76.0)		
	Unmarried/divorced/widowed	15 (30.0)	12 (24.0)		
AD duration (years)				0.068	0.946
	Range	1–8	0.6–9		
	Mean ± SD	4.28 ± 1.51	4.30 ± 1.45		
OCD duration (years)				0.466	0.642
	Range	0.5–7	0.6–8		
	Mean ± SD	3.92 ± 1.38	3.79 ± 1.41		
Severity of AD				0.832	0.660
	Mild	16 (32.0)	17 (34.0)		
	Moderate	20 (40.0)	18 (36.0)		
	Severe	14 (28.0)	15 (30.0)		
Education level				0.412	0.814
	Primary school	9 (18.0)	8 (16.0)		
	Junior high/high school	26 (52.0)	24 (48.0)		
	College or above	15 (30.0)	18 (36.0)		
Employment status				0.649	0.723
	Employed	32 (64.0)	34 (68.0)		
	Retired	8 (16.0)	9 (18.0)		
	Unemployed	10 (20.0)	7 (14.0)		

Note: AD, atopic dermatitis; OCD, obsessive-compulsive disorder; MCBT, mobile application-based cognitive behavioural therapy; SD, standard deviation.

### Comparison of EASI Scores Before and After Treatment Between the Two Groups

At baseline, EASI scores did not differ significantly between groups (*p* > 0.05). After 12 weeks, both groups showed significant reductions from baseline (conventional group Z = –2.868, *p* = 0.004; MCBT group Z = –3.729, *p* < 0.001). The MCBT group had significantly lower EASI scores than the conventional group (Z = –2.475, *p* = 0.008, Table 2).

**Table 2.** **Comparison of EASI scores before and after treatment in the two groups [M (Q1, Q3)]**.

Group	Before treatment	After treatment	Z^a^	*p* ^a^
Conventional group (n = 50)	18.5 (9.2, 33.9)	14.4 (6.8, 26.9)	–2.868	0.004
MCBT group (n = 50)	19.5 (8.4, 34.3)	10.5 (3.7, 18.6)	–3.729	<0.001
Z^b^	0.365	–2.475		
*p* ^b^	0.717	0.008		

Note: EASI, Eczema Area and Severity Index; MCBT, mobile application-based cognitive behavioural therapy; ^a^, Wilcoxon signed-rank test for within-group comparisons; ^b^, Mann–Whitney U test for between-group comparisons; M (Q1, Q3), median and interquartile range.

### Comparison of Y-BOCS Scores Before and After Treatment in the Two Groups

No significant difference was observed in Y-BOCS scores between groups at baseline (*p* > 0.05). After 12 weeks, both groups showed significant reductions compared with baseline (conventional group t = 11.481, *p* < 0.001; MCBT group t = 21.208, *p* < 0.001), and the MCBT group had significantly lower scores than the conventional group (t = 12.033, *p* < 0.001, Table 3).

**Table 3.** **Comparison of Y-BOCS scores before and after treatment in the two groups (mean ± SD)**.

Group	Before treatment	After treatment	t	*p*
Conventional group (n = 50)	22.3 ± 3.5	15.5 ± 2.3	11.481	<0.001
MCBT group (n = 50)	22.7 ± 3.6	10.2 ± 2.1	21.208	<0.001
t	0.563	12.033		
*p*	0.575	<0.001		

Note: Y-BOCS, the Yale–Brown Obsessive Compulsive Scale; MCBT, mobile application-based cognitive behavioural therapy; SD, standard deviation.

### Comparison of DLQI Scores Before and After Treatment in the Two Groups

No significant difference was observed in DLQI scores between groups at baseline (*p* > 0.05). After 12 weeks, both groups showed significant reductions compared with baseline (conventional group t = 11.188, *p* < 0.001; MCBT group t = 21.319, *p* < 0.001), and the MCBT group showed significantly lower scores than the conventional group (t = 11.549, *p* < 0.001, Table 4).

**Table 4.** **Comparison of DLQI scores before and after treatment in the two groups (mean ± SD)**.

Group	Before treatment	After treatment	t	*p*
Conventional group (n = 50)	18.5 ± 4.2	10.6 ± 2.7	11.188	<0.001
MCBT group (n = 50)	18.8 ± 4.1	5.3 ± 1.8	21.319	<0.001
t	0.361	11.549		
*p*	0.719	<0.001		

Note: DLQI, the Dermatology Life Quality Index; MCBT, mobile application-based cognitive behavioural therapy; SD, standard deviation.

### Comparison of Treatment Adherence Between the Two Groups

After 12 weeks, the overall adherence rate was significantly higher in the MCBT group (82.0%) than that in the conventional group (64.0%) (χ^2^ = 4.110, *p* = 0.042, Table 5).

**Table 5.** **Comparison of treatment adherence between the two groups [n (%)]**.

Group	High adherence	Moderate adherence	Low adherence	Overall adherence	χ^2^	*p*
Conventional group (n = 50)	17 (34.0)	15 (30.0)	18 (36.0)	32 (64.0)	4.110	0.042
MCBT group (n = 50)	24 (48.0)	17 (34.0)	9 (18.0)	41 (82.0)		

Note: MCBT, mobile application-based cognitive behavioural therapy.

## Discussion

Persistent skin lesions in patients with AD can lead to OCD, and compulsive thoughts and behaviours (e.g., compulsive cleaning and repeated scratching) can exacerbate skin lesions and itching, forming a vicious cycle. The recent “brain–cortex axis” hypothesis suggests a complex interaction between the cerebral cortex and the skin, and chronic dermatitis and mental disorders are very common comorbidities [16]. Therefore, psychotherapy is of great importance for AD patients with OCD. Although conventional CBT can improve patients’ disease perception and help them understand the harm of bad behaviour, patients usually receive treatment at home and have poor adherence. Hollmann *et al*. [17] reported that, compared with conventional patient education, smartphone application-based CBT improved acceptance and satisfaction in patients with OCD. A study conducted by Akin-Sari *et al*. [18] during the COVID-19 pandemic confirmed that using mobile applications to deliver CBT for patients with OCD in the community effectively reduced OCD symptoms and improve cognitive function.

In this study, the MCBT group received remote CBT via mobile applications, which significantly improved patient treatment adherence and reduced EASI, Y-BOCS, and DLQI scores. The reason is that, compared with conventional CBT, MCBT leverages mobile applications (e.g., WeChat official account, mini-program, and chat groups) to deliver remote real-time therapy, thereby effectively improving the efficiency of doctor–patient communication. Through multimedia materials on these platforms, patients can access disease education at any time, effectively improving their cognitive level and correcting bad behaviours. A scoping review suggested that the optimal way to provide health services to people with mental illnesses using mobile applications is under the guidance of a therapist [19]. In this study, the MCBT group received continuous home-based treatment. Through online clinics and video call functions in WeChat groups, physicians can conduct online diagnoses, help patients establish reasonable cognitive patterns, promptly identify and correct patients’ bad behaviours, and improve treatment adherence. They can also answer patients’ questions at any time, making it easier for them to accurately implement CBT techniques such as attention distraction, meditation, and alternative behaviours, which more effectively reduced compulsive behaviours (e.g., scratching and picking) and relieve skin symptoms, thereby improving their quality of life. Zhai *et al*. [20] reported that 12 weeks of internet-based CBT in patients with AD reduced levels of mood-related neurotransmitters and alleviated dermatitis symptoms. Barbieri *et al*. [21] also confirmed the combined efficacy of MCBT on skin and psychological symptoms in patients with psoriasis and depression. Furthermore, patients can exchange rehabilitation experiences in the WeChat group, which not only facilitates experience sharing but also improved social skills, reduced feelings of inferiority, and build confidence in recovery. During the consolidation and relapse prevention stage, patients can continue to receive antiallergy treatment and healthy lifestyle guidance to prevent disease relapse.

This study has certain limitations:

Firstly, this was a retrospective single-centre study with a relatively small sample size, which may introduce bias and residual confounding and limits causal inference between MCBT and clinical outcomes. Therefore, the findings should be interpreted cautiously. Secondly, the follow-up period was short, and long-term treatment effects and recurrence rates were not observed. Thirdly, due to the retrospective design, blinding was not implemented, which may have introduced assessment bias. Fourthly, although baseline dermatological and psychological severity (e.g., EASI, Y-BOCS, and DLQI scores) were comparable between groups, some potential confounding factors could not be fully controlled. Moreover, detailed socioeconomic indicators (e.g., income level) were not collected; employment status was used as a surrogate socioeconomic variable, but residual confounding may still exist. Finally, treatment outcomes may also have been influenced by factors such as therapist experience and patient cognitive level.

Future prospective studies or randomized controlled trials are needed to further validate the efficacy of MCBT. In addition, future research could expand the sample size, extend the follow-up period, conduct multicentre studies, analyse confounding factors, and optimize treatment regimens.

## Conclusions

MCBT combined with conventional therapy may help alleviate dermatological and obsessive-compulsive symptoms in patients with AD and OCD, while also enhancing treatment adherence and quality of life. Given its simplicity and feasibility, further large-scale prospective studies are warranted to validate its clinical value.

## Availability of Data and Materials

The datasets used and/or analysed during the current study were available from the corresponding author on reasonable request.
